# Characterization of induced tissue-specific stem cells from pancreas by a synthetic self-replicative RNA

**DOI:** 10.1038/s41598-018-30784-0

**Published:** 2018-08-17

**Authors:** Chika Miyagi-Shiohira, Yoshiki Nakashima, Naoya Kobayashi, Issei Saitoh, Masami Watanabe, Hirofumi Noguchi

**Affiliations:** 1 Department of Regenerative Medicine, Graduate School of Medicine, University of the Ryukyus, Okinawa, 903-0215 Japan; 2 Okayama Saidaiji Hospital, Okayama, 704-8192 Japan; 3 Division of Pediatric Dentistry, Graduate School of Medical and Dental Science, Niigata University, Niigata, 951-8514 Japan; 4 Department of Urology, Okayama University Graduate School of Medicine, Dentistry and Pharmaceutical Sciences, Okayama, 700-8558 Japan

## Abstract

Induced pluripotent stem (iPS) cells have significant implications for overcoming most of the ethical issues associated with embryonic stem (ES) cells. Furthermore, our recent study demonstrated the generation of induced tissue-specific stem (iTS) cells by transient overexpression of the reprogramming factors using a plasmid combined with tissue-specific selection. In this study, we were able to generate RNA-based iTS cells that utilize a single, synthetic, self-replicating VEE-RF RNA replicon expressing four reprogramming factors (OCT4, KLF4, SOX2, and GLIS1). A single VEE-RF RNA transfection into mouse pancreatic tissue resulted in efficient generation of iTS cells from pancreas (iTS-P cells) with genetic markers of endoderm and pancreatic progenitors and differentiation into insulin-producing cells more efficiently than ES cells. Subcutaneous transplantation of iTS-P cells into immunodeficient mice resulted in no teratoma formation. Bisulfite genomic sequencing demonstrated that the promoters of Oct4 and Nanog remained partially methylated in iTS-P cells. We compared the global gene-expression profiles of ES cells, iTS-P cells, and pancreatic islets. Microarray analyses confirmed that the iTS-P cells were similar but not identical to ES cells compared with islets. These data suggest that iTS-P cells are cells that inherit numerous components of epigenetic memory from pancreas cells and acquire self-renewal potential. The generation of iTS cells may have important implications for the clinical application of stem cells.

## Introduction

Embryonic stem (ES) cells and induced pluripotent stem (iPS) cells are capable of unlimited proliferation *in vitro* while maintaining their potential to differentiate into cells from the three embryonic germ layers^1–7^. The generation of iPS cells without the genomic integration of exogenous reprogramming factors by plasmids^8–10^ and adenoviruses^11^ has been reported. Recently, a single, synthetic, self-replicating VEE-RF RNA replicon expressing four reprogramming factors (OCT4, KLF4, SOX2, and GLIS1) at consistently high levels prior to regulated RNA degradation was utilized to generate iPS cells^12^. The production of iPS cells without insertional mutagenesis addresses a critical safety concern for the potential use of iPS cells in regenerative medicine. However, the use of iPS cells for clinical therapies is hampered by their potential for tumor formation and the limited ability to generate pure populations of differentiated cell types *in vitro*.

Recently, we have focused on developing a method for generating induced tissue-specific stem (iTS) cells derived from pancreas (iTS-P) or liver (ITS-L) by transfection with a plasmid harboring cDNAs for Oct3/4, Sox2, Klf4, and c-Myc and subsequent tissue-specific selection^13,14^. Notably, they were unable to generate teratomas when transplanted subcutaneously into immunodeficient mice. They expressed several genetic markers for endoderm and pancreatic/hepatic progenitors and differentiated into insulin-producing cells/hepatocytes more frequently than ES cells upon differentiation induction. It has recently been shown that, following the reprogramming of mouse/human iPS cells, epigenetic memory is inherited from the parental cells^15–20^. These findings suggest that the phenotype of iPS cells may be influenced by their cells of origin and that their skewed differentiation potential may prove useful in the generation of differentiated cell types that are currently hard to produce from ES/iPS cells for the treatment of human diseases. iTS cells must therefore be cells that inherit numerous components of epigenetic memory from pancreas/liver cells and acquire self-renewal potential.

In this study, we generated iTS cells from mouse pancreatic tissue (iTS-P) using a single, synthetic, self-replicating VEE-RF RNA replicon expressing four reprogramming factors (OCT4, KLF4, SOX2, and GLIS1). Since plasmid transfection was used for the generation of iTS cells in our previous studies and 10–20% of DNA integration of the plasmid, an RNA vector was used in this study to avoid potential integration problems. We also characterized the iTS-P cells derived from the RNA vector compared with pancreatic islets and ES cells by methylation and microarray analyses.

## Results

### Generation of iTS-P cells from mouse pancreatic tissue by RNA vector

**Figure 1:**
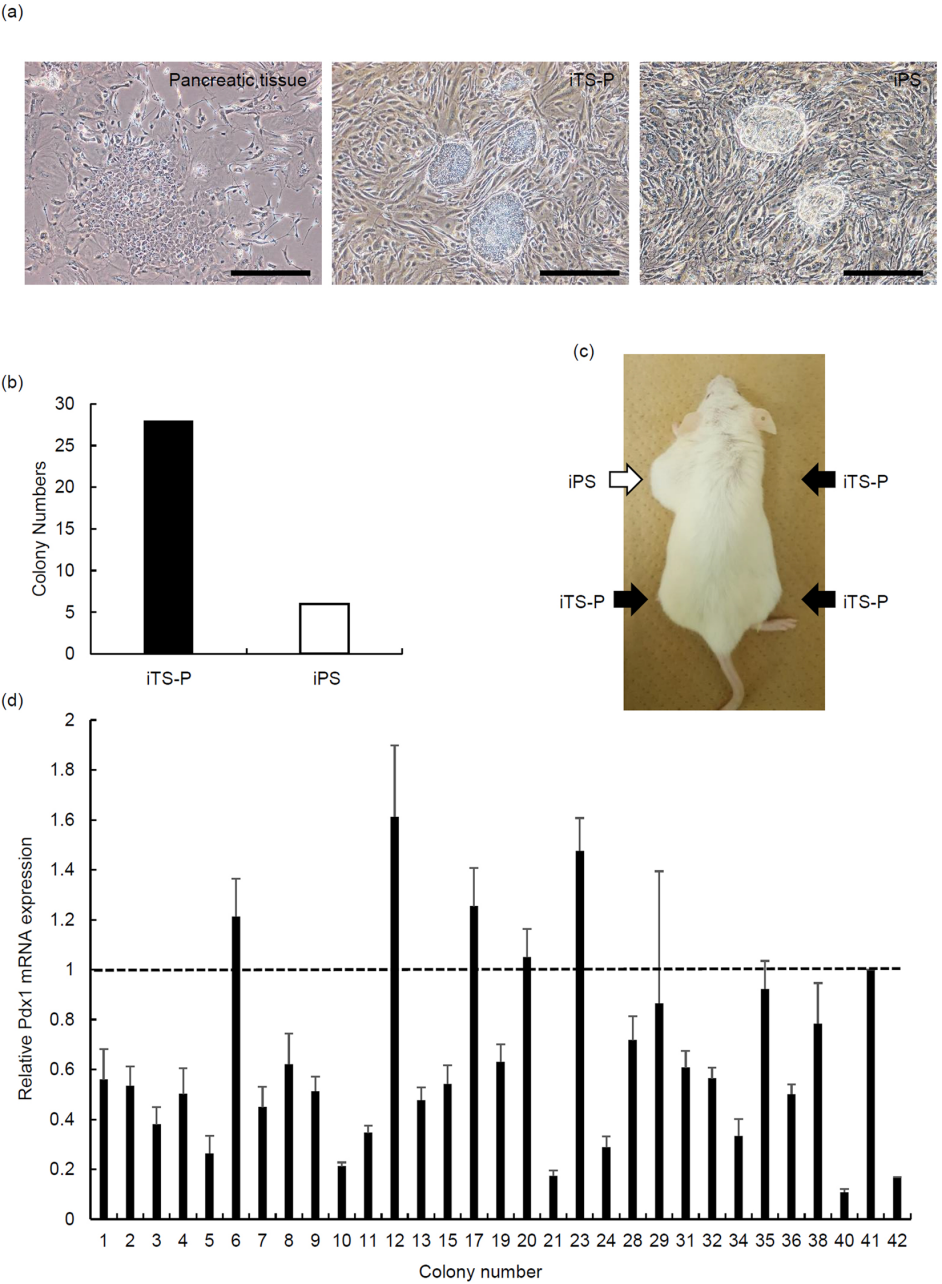
Generation of iTS-P cells by a synthetic self-replicative RNA. (**a**) The morphology of mouse pancreatic tissue, iTS-P cells, and iPS cells. Scale bars = 200 µm. (**b**) The colony numbers of iTS-P and iPS cells. The synthetic self-replicative RNA was transfected into pancreatic tissue from C57/BL6 mouse at 24 weeks of age, and the number of colonies was counted after 23–45 days. (**c**) Teratoma formation/tumorigenicity assay. A total of 1 × 10^6^ to 1 × 10^7^ iPS/iTS cells were inoculated into each humerus and thigh of NOD/scid mice. (**d**) The quantitative RT-PCR analysis of Pdx1 genes, markers of pancreatic stem/progenitor cells, in iTS-P cells. A total of 28 iTS-P clones were evaluated for their expression of Pdx1 by quantitative RT-PCR. #5, 7, 10 clones were iPS cells. The data are expressed as the Pdx1-to-Gapdh ratio, with the ratio of the #41 clone arbitrarily set at 1 (n = 5). Error bars represent the standard error.

We attempted to generate mouse iTS cells from 24-week-old donor pancreata by transfection of a single, synthetic, self-replicating VEE-RF RNA replicon expressing four reprogramming factors (OCT4, KLF4, SOX2, and GLIS1) at consistently high levels prior to regulated RNA degradation (Supplemental Fig. 1a). We were able to generate 42 colonies of iPS/iTS-P like morphology from 24-week-old mouse pancreata using the RNA vector. Thirty-four clones of the 42 colonies had self-renewing potential (Fig. 1a). Six of the 34 clones showed an iPS-like morphology and were able to generate teratoma formation (Fig. 1b,c, Table 1). Twenty-eight of the 34 clones showed a pancreatic stem cell-like (iTS-P-like) morphology and were unable to generate teratoma formation (Fig. 1b,c, Table 1). These 28 clones were evaluated for their expression of Pdx1, the marker of pancreatic stem/progenitor cells. All of these clones expressed Pdx1 mRNA (Fig. 1d). Since the expression of Pdx1 in clones #6, 12, 17, 20, 23, and 41 was higher than in other clones, we used these clones for subsequent experiments.

**Table 1:** Teratoma formation.

Cell Type	Injected cell number	Mice bearing teratoma/total mice injected	Period (days)
ES	1 × 10^6^	5/5	60
iPS	1 × 10^6^	6/6	60
iTS-P	1 × 10^6^	0/28	160

### Characterization of iTS-P cells by RNA vector

**Figure 2:**
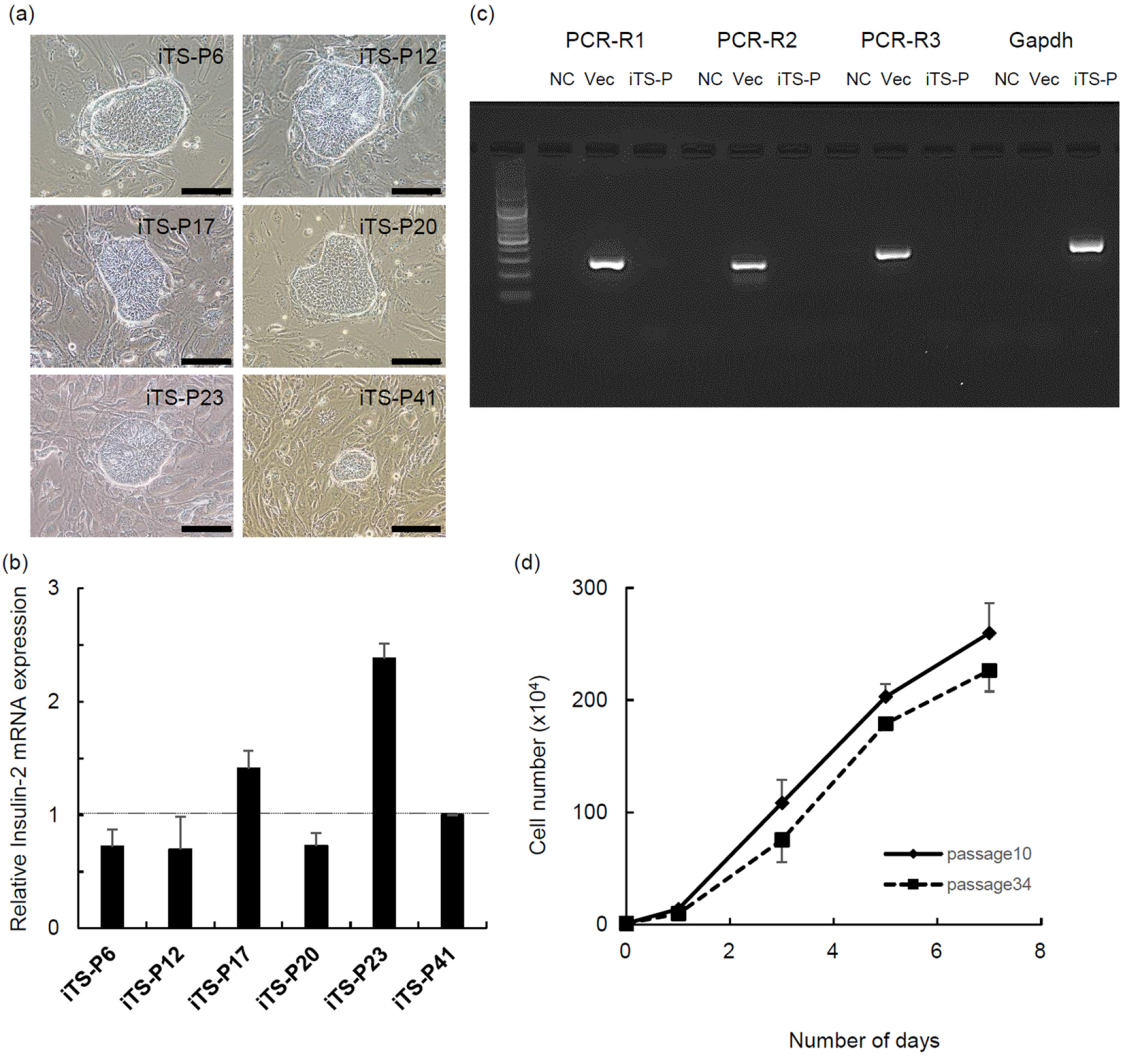
Characterization of iTS-P cells. (**a**) The morphology of iTS-P clones in which the expression of Pdx1 was higher than in other clones. Scale bars = 100 µm. (**b**) The quantitative RT-PCR analysis of insulin-2 genes in differentiated iTS-P cells. Differentiated cells derived from iTS-P cells (passage 20) by stage 4–5 were analyzed by quantitative RT-PCR. The data are expressed as the insulin-2-to-Gapdh ratio, with the ratio of the #41 clone arbitrarily set at 1 (n = 5). Error bars represent the standard error. (**c**) The RT-PCR analysis for persistent VEE-RF RNA Replicon in iTS-P23 cells. Total RNA from iTS-P23 cells was prepared from passage 8. No template was used as a negative control (NC). The VEE-RF RNA Replicon itself was used as a positive control (Vec). Size: R1 = 302 bp, R2 = 302 bp, R3 = 394 bp, Gapdh = 452 bp (**d**) Growth curves of iTS-P23 cells (passages 10 and 34). Error bars represent the standard error.

We applied a stepwise differentiation protocol to evaluate the six clones (Fig. 2a) with a higher Pdx1 mRNA expression than other clones that were to be differentiated into insulin-producing cells^21,22^. Since iTS-P cells express endodermal cell markers, we included stages 4 and 5 of the induction protocol in the stepwise differentiation protocol. All six clones induced the expression of insulin-2 mRNA (Fig. 2b). A reverse transcription-polymerase chain reaction (RT-PCR) analysis of isolated RNAs showed that the iTS-P23 cells expressing the highest insulin-2 mRNA after treatment of induction medium had lost the VEE-RF-RNA replicon by passage 8 (Fig. 2c). The iTS-P23 cells continued to divide actively beyond passage 30 without changes in their morphology or growth activity (Fig. 2d).

### Gene expression of iTS-P cells

**Figure 3:**
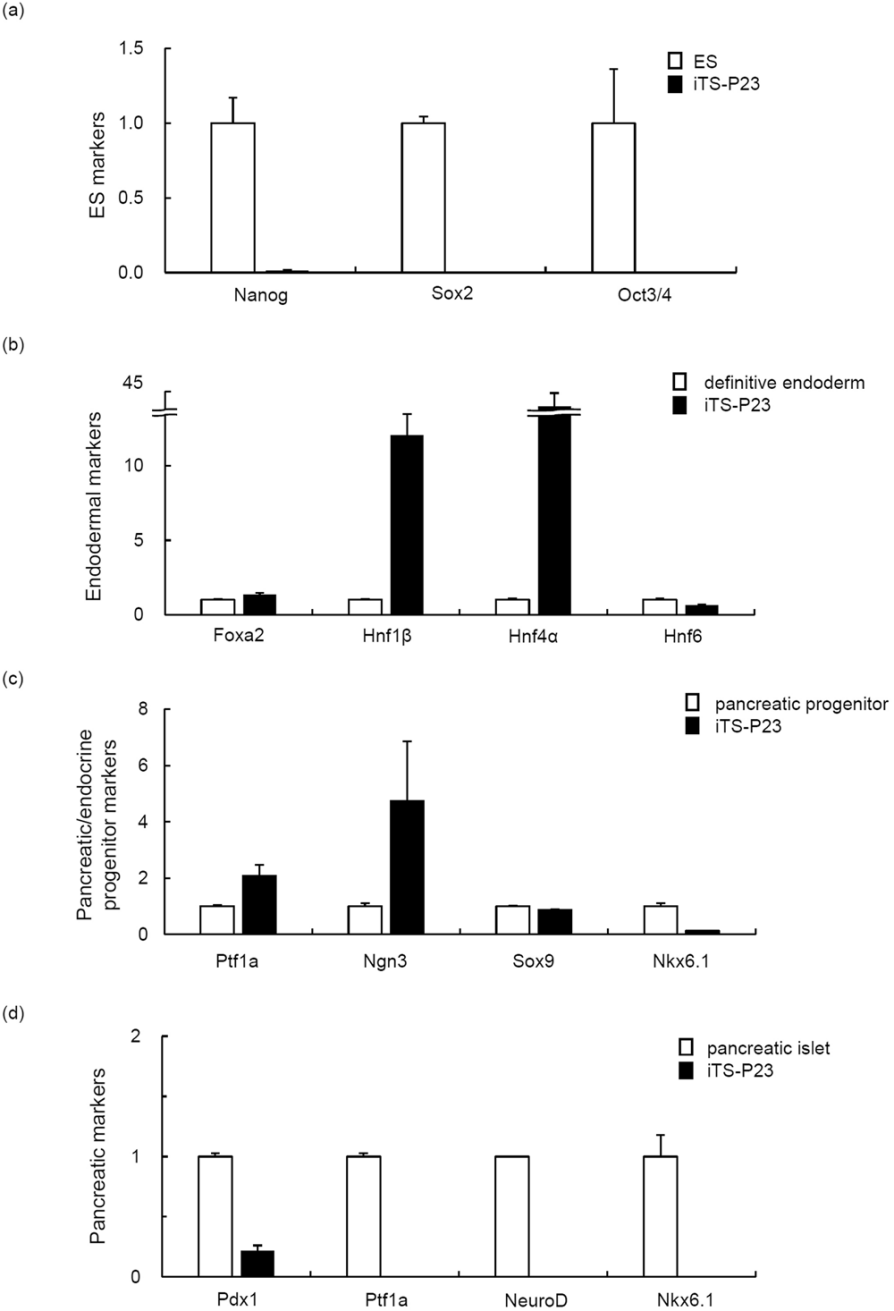
A quantitative RT-PCR analysis of ES and endodermal/pancreatic cell marker genes in iTS-P23 cells. (**a**) The quantitative RT-PCR analysis of ES cell marker genes in iTS-P23 cells. ES cells were used as a control. (**b**) The quantitative RT-PCR analysis of endodermal cell marker genes in iTS-P23 cells. Definitive endoderm cells differentiated from ES cells (stage 1–3) were used as a control. (**c**) The quantitative RT-PCR to detect pancreatic/endocrine progenitor cell marker genes in iTS-P23 cells. Pancreatic/endocrine progenitor cells (cells isolated from E10–14 mouse embryos) were used as a control. (**d**) The quantitative RT-PCR analysis of pancreatic cell marker genes in iTS-P cells. Pancreatic cells (<90% islets) cells were used as a control. The data are expressed as the genes-to-Gapdh ratio, with the ratio of the control cells arbitrarily set at 1 (n = 5). Error bars represent the standard error.

To investigate the gene expression in iTS-P23 cells, an RT-PCR analysis of ES cell marker genes was performed. The expression of the pluripotency markers Nanog, Sox2, and Oct3/4 in iTS-P23 cells was significantly lower than that in ES cells (Fig. 3a). We next investigated the gene expression patterns of endodermal markers. Cells differentiated from ES cells (generated by a stepwise differentiation protocol that relies on intermediates thought to be similar to the cell populations present in the developing embryo; Stage 1–3)^21,22^ were used as a positive control. The expression of endodermal marker genes (forkhead box protein a2; Foxa2, hepatocyte nuclear factor 1β, 4α, 6; Hnf1β, 4α, 6) was detected in iTS-P23 cells (Fig. 3b), which was similar to the patterns detected in our mouse pancreatic stem cell line HN#13 and in the iTS-P cells we previously reported^13,14,23^, but not ES cells. We next investigated the gene expression patterns of pancreatic/endocrine progenitor (PP) markers. Cells isolated from E10-14 mouse embryos were used as a positive control. The expression of pancreatic/endocrine progenitor marker genes (pancreas transcription factor 1 subunit alpha; Ptf1A, Neurogenin-3; Ngn3, Sox9) was detected in PP cells. The expression of Sox9 gene was detected in iTS-P23 cells, whereas the expression of Ptf1a, Ngn3, or Nkx6.1 genes were not strongly detected in the cells (Fig. 3c). We next investigated the gene expression patterns of pancreatic markers. Pancreatic islets were used as a positive control. The expression of Pdx1 gene was detected in iTS-P23 cells, whereas the expression of Ptf1a, NeuroD, or Nkx6.1 genes were not strongly detected in the cells (Fig. 3d).

### Differentiation of iTS-P cells into insulin-producing cells

**Figure 4:**
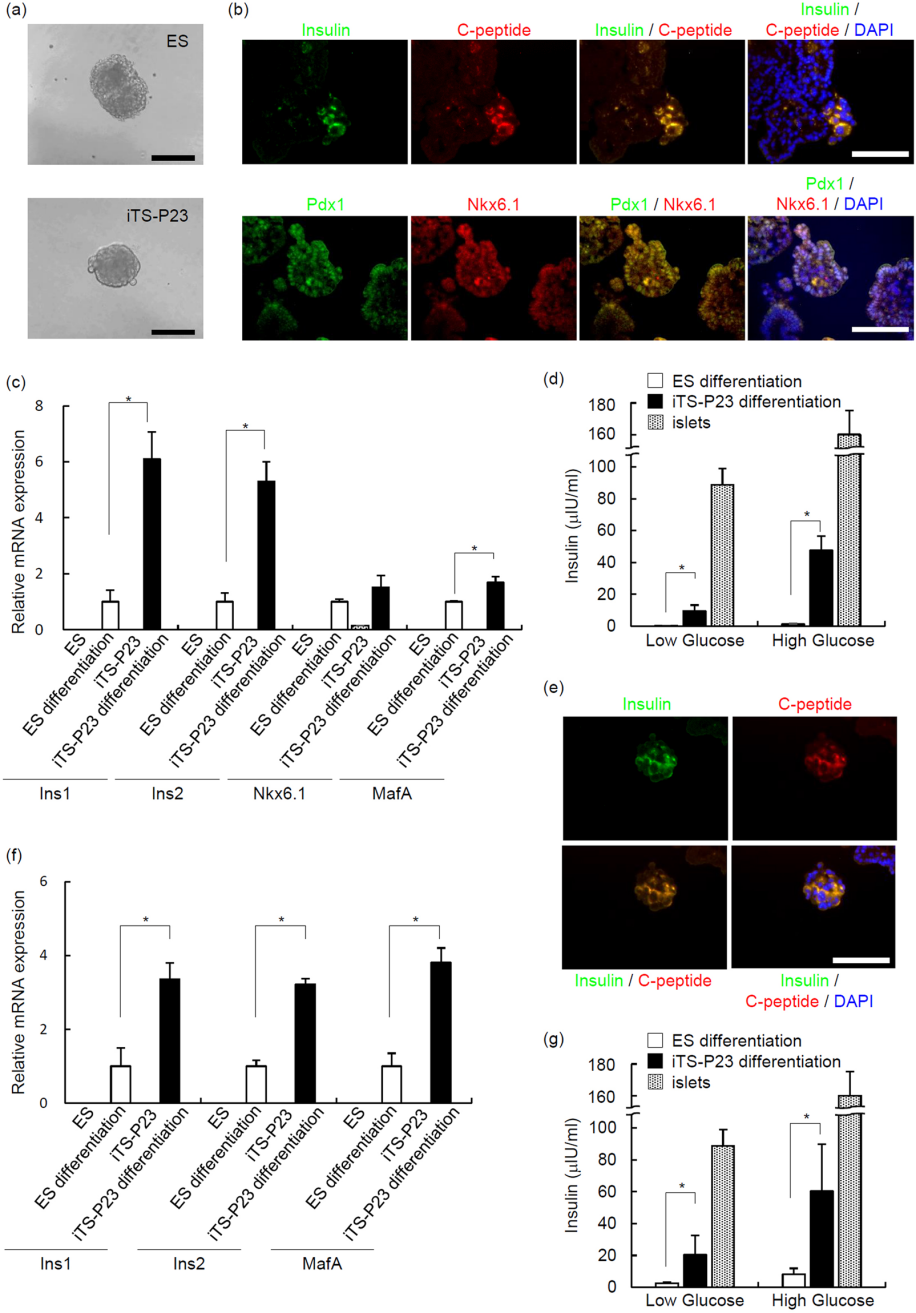
Differentiation of iTS-P23 cells into insulin-producing cells. (**a**) EB/spheroid formation. The differentiation of ES/iTS cells into insulin-producing cells was also conducted by EB/spheroid formation. EBs/spheroids were allowed to undergo spontaneous differentiation for seven days in suspension. Scale bars = 200 µm. (**b**) Immunostaining of insulin-producing cells derived from iTS-P cells (insulin, C-peptide, Pdx1, and Nkx6.1). Scale bars = 100 µm. (**c**) The expression levels insulin-1, insulin-2, Nkx6.1, and MafA transcripts in day 7 EBs/spheroids analyzed by quantitative RT-PCR. The data are expressed as the genes-to-Gapdh ratio, with the ratio of the EBs derived from ES cells arbitrarily set at 1 (n = 5). Error bars represent the standard error. (**d**) The insulin release assay. Differentiated iTS-P and ES cells were stimulated with 2.8 and 20 mM D-glucose, and the amount of insulin released into the culture supernatant was analyzed by ELISA. (**e**) Immunostaining of insulin-producing cells derived from iTS-P cells by a stepwise differentiation protocol (insulin and C-peptide). Scale bars = 100 µm. (**f**) The expression levels of insulin-1, insulin-2, and MafA transcripts in differentiated iTS-P cells were analyzed by a quantitative RT-PCR. The data are expressed as the genes-to-Gapdh ratio, with the ratio of the differentiated ES cells arbitrarily set at 1 (n = 5). Error bars represent the standard error. (**g**) The insulin release assay. Differentiated iTS-P and ES cells were stimulated with 2.8 and 20 mM D-glucose, and the amount of insulin released into the culture supernatant was analyzed by ELISA. Error bars represent the standard error. *p < 0.05.

To address the possibility that iTS-P cells were prone to skewed differentiation into insulin-producing cells, the expression of insulin-1 and insulin-2 genes and proteins was analyzed in spheroids derived from iTS-P cells and compared to embryonic bodies (EBs) in ES cells (Fig. 4a). The spheroids from iTS-P23 cells differentiated into insulin-producing cells (Fig. 4b) more efficiently than EBs from ES cells (Fig. 4c). The insulin-positive cells were C-peptide-positive (Fig. 4b), thus excluding the possibility of the uptake of insulin from the media; 5.56 ± 0.68% of the differentiated cells were insulin/C-peptide-positive (n = 10), while the percentage of insulin/C-peptide-positive cells differentiated from ES cells was <1% (n = 10). This skewed differentiation manifests as their increased capacity for spontaneous differentiation into insulin-producing cells. The differentiated iTS-P cells expressed Pdx1 and Nkx6.1 proteins (Fig. 4b) and Nkx6.1 and MafA mRNA (Fig. 4c). To evaluate whether the differentiated cells had glucose sensitivity, the differentiated cells from the iTS-P cells were exposed to low (2.8 mM) or high (20 mM) concentrations of glucose. The amount of insulin released by the cells was approximately 10–20 fold higher than that released by an ES-derived population at both glucose concentrations, although the amount of insulin was lower than that of islets (Fig. 4d).

We applied a direct differentiation method (stepwise differentiation protocol^21,22^) to drive the generation of insulin-expressing cells. Using the method, iTS-P23 cells efficiently differentiated into insulin-producing cells (insulin/Cpeptide-positive cells; 6.87 ± 2.15%, n = 8; Fig. 4e). The differentiated iTS-P cells expressed insulin-1, -2 and MafA mRNA (Fig. 4f). The amount of insulin released by the differentiated iTS-P cells was higher than that released by an ES-derived population at both glucose concentrations (Fig. 4g).

### Bisulfite genomic sequencing of the promoter regions of Oct3/4, Nanog, Insulin-1, and Insulin-2 in iTS-P and ES cells

**Figure 5:**
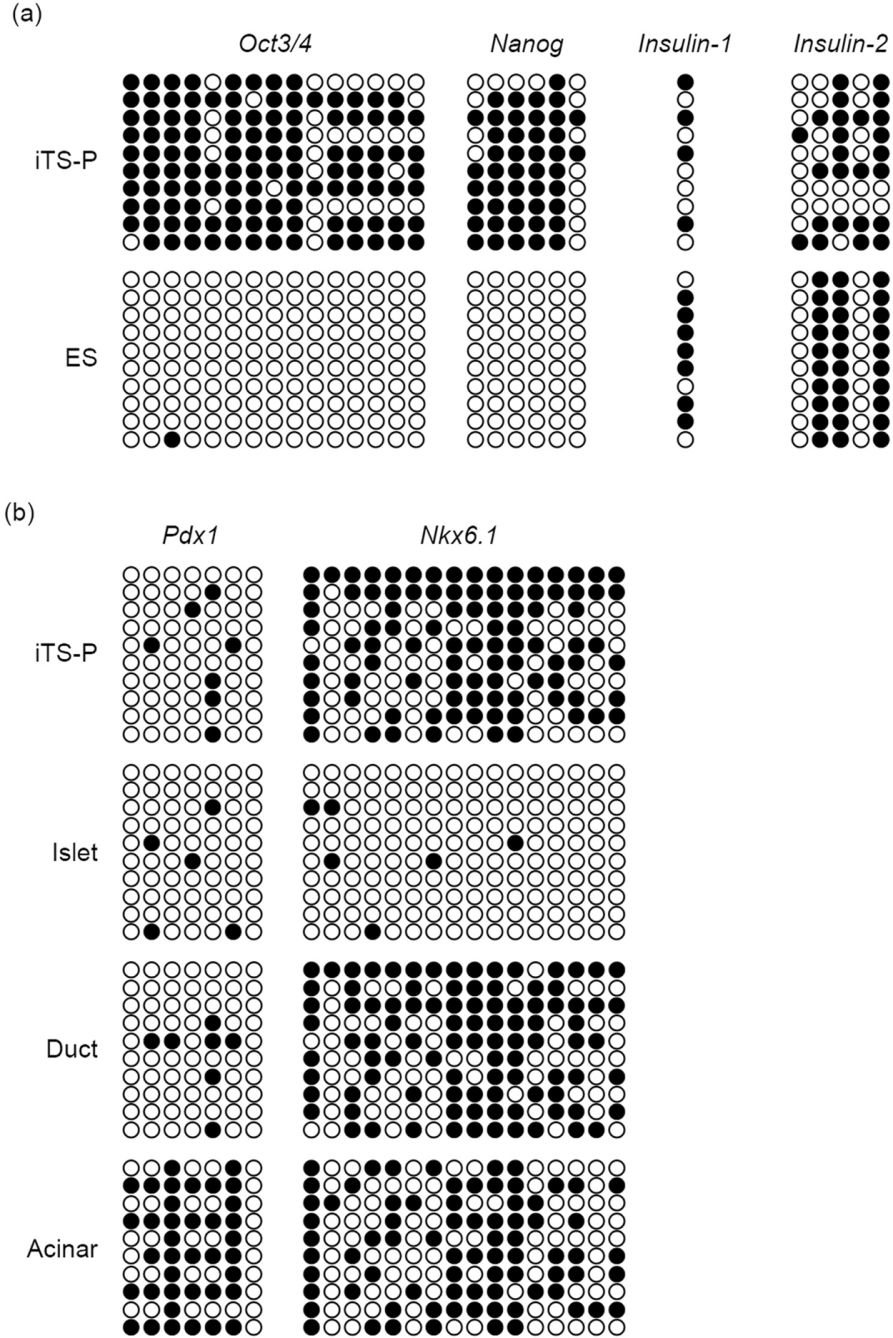
Bisulfite genomic sequencing. (**a**) Bisulfite genomic sequencing of the promoter regions of Oct3/4, Nanog, insulin-1, and insulin-2 in iTS-P23 cells and ES cells. (**b**) Bisulfite genomic sequencing of the promoter regions of Pdx1 and Nkx6.1 in iTS-P23 cells, islet cells, duct cells, and acinar cells. Open circles indicate unmethylated CpG dinucleotides, while closed circles indicate methylated CpGs.

Bisulfite genomic sequencing demonstrated that the promoters of Oct3/4 and Nanog remained methylated in iTS-P cells but were demethylated in ES cells. In contrast, the promoters of insulin-1 and insulin-2 were methylated in both iTS-P and ES cells, although the rate of methylation was higher in ES cells than in iTS-P cells (Fig. 5a). These results demonstrate that methylation of the promoters in iTS-P cells is not similar to that in ES cells. We also evaluated the promoters of Pdx1 and Nkx6.1. The promoter of Pdx1 was demethylated and that of Nkx6.1 was methylated in iTS-P cells. On the other hand, the promoters of both Pdx1 and Nkx6.1 were demethylated in pancreatic islets and were methylated in acinar cells. In ductal cells, the promoter of Pdx1 was demethylated and that of Nkx6.1 was methylated in iTS-P cells (Fig. 5b). These results demonstrate that methylation pattern of Pdx1 and Nkx6.1 promoters in iTS-P cells is similar to that in ductal cells.

### Microarray data

**Figure 6:**
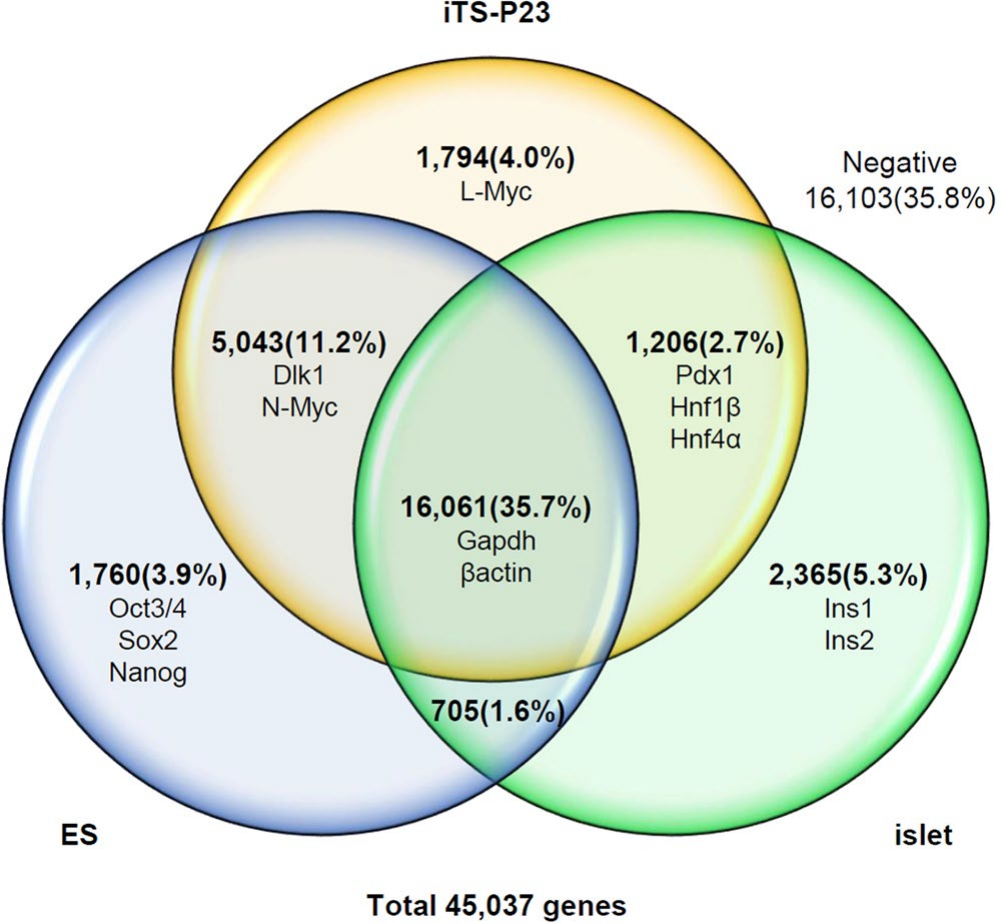
Microarray. Total RNA from ES cells, iTS-P cells, or islets was labeled with biotin. Samples were hybridized to the GeneChip 3′IVT PLUS Reagent Kit (Affymetrix) and CeneChip Hybridization, Wash and Stain Kit (Affymetrix) according to the manufacturer’s protocol. Arrays were scanned with a GeneChip Scanner 3000 7G (Affymetrix). Data were analyzed using the Affymetrix GeneChip Command Console software program (Affymetrix).

We compared the global gene-expression profiles of ES cells, iTS-P cells, and pancreatic islets using microarrays (Fig. 6). A total of 45,037 genes were evaluated, and 28,934 genes (64.2%) were positive in ES cells, iTS-P cells, and/or pancreatic islets, while 16,103 genes (35.8%) were negative in these cells. A total of 1,760 genes (3.9%), including Oct3/4, Sox2, and Nanog, were positive in only ES cells; 1,794 genes (4.0%), including L-Myc, were positive in only iTS-P cells; 2,365 genes (5, 3%), including insulin-1 and insulin-2, were positive in only pancreatic islets; 5,043 genes (11.2%), including Dlk1 and N-Myc, were positive in ES cells and iTS-P cells; and 1,206 genes (2.7%), including Pdx1, Hnf1β, and Hnf4α, were positive in iTS-P cells and pancreatic islets. The analysis revealed that iTS-P cells are clustered more closely with ES cells than pancreatic islets, although iTS-P cells are markedly different from ES cells. A total of 16,061 genes (35.7%), including Gapdh and β-actin, were positive in all 3 cell types.

## Discussion

Cell replacement therapies, such as transplantation of purified islets, have emerged as promising alternatives to whole-organ transplantation for the treatment of patients with type 1 diabetes^24,25^. These observations are proof-of-concept and have intensified interest in treating diabetes not only by cell transplantation but also by stem cells. The regeneration of β cells from stem and progenitor cells is an attractive method of restoring the islet cell mass. Protocols for the *in vitro* differentiation of ES/iPS cells based on normal developmental processes have generated β-like cells that produce high levels of insulin^21,22,26^, albeit at low efficiency and without full responsiveness to extracellular levels of glucose. Although pancreatic stem/progenitor cells have been identified^23,27–32^, pancreatic “progenitor” cells have limited self-renewal capacity, and it is extremely difficult to isolate human pancreatic “stem” cells with self-renewal capacity^33^. Therefore, the generation of iTS-P cells using iPS-cell technology may create several possibilities for the development of new treatments for diabetes.

The iTS-P cells were able to differentiate into insulin-producing cells more efficiently than ES cells. Furthermore, the iTS-P cells do not form teratomas. ES/iPS cells carry a risk of teratoma formation, even after transplantation of differentiated cells derived from ES/iPS cells, due to possible contamination with undifferentiated cells. This is one of the advantages of iTS-P cells over ES/iPS cells in terms of potential clinical use. Bisulfite genomic sequencing in this study clearly demonstrated that the promoters of Oct3/4 and Nanog remained methylated in iTS-P cells, while the promoters were demethylated in ES cells. Moreover, quantitative RT-PCR showed that there were few expressions of Oct3/4 or Nanog. These results demonstrate that methylation of the promoters in iTS-P cells is not similar to that in ES cells; therefore, iTS-P cells are unlikely to have pluripotency or teratoma formation.

The global gene-expression profiles of ES cells, iTS-P cells, and pancreatic islets using microarrays showed that iTS-P cells were markedly different from iPS cells and pancreatic islets. Of the 45,037 total genes evaluated, 11.2% were positive in both ES cells and iTS-P cells, while 2.7% were positive in both iTS-P cells and pancreatic islets, showing that iTS-P cells were more closely related to ES cells than pancreatic islets. Interestingly, L-Myc was positive in only iTS-P cells, while c-Myc and N-Myc were positive in both ES cells and iTS-P cells. The Myc family of transcription factors comprises c-Myc, N-Myc, and L-Myc and has been implicated in the generation of a variety of human tumors. It has been reported that *L-myc* knockout mice develop normally^33^, embryos lacking *c-myc* die before E10.5 due to hematopoietic and placental defects^34,35^, and *N-myc*-deficient embryos die before E11.5 due to neuroectodermal and heart defects^36^. Myc activity is essential for efficient cellular reprogramming^37^ and has complex roles in various stem and progenitor cell types^38^. A recent study showed that Myc controls the biosynthetic machinery of stem cells without affecting their potency^39^. Differences in the L-Myc expression between ES cells and iTS-P cells may be an important factor influencing differences in the characteristics of ES cells and iTS-P cells.

The expression of Pdx1 in clones #6, 12, 17, 20, 23, and 41 was higher than that in the other clones (Fig. 1d) and the expression of insulin-2 after their differentiation differed among the clones (Fig. 2b). Our previous study also showed that the differentiation ability of iTS-P cells into insulin-producing cells depends on the individual clone^13^. Since Yamanaka’s group showed the generation of germline-competent iPS cells via the selection for the expression of *Nanog* instead of *Fbx15*, the method of clone selection may be one of key factors for generating high quality iTS-P cells.

In conclusion, we generated iTS cells from mouse pancreas cells using a single, synthetic, self-replicating RNA vector expressing four reprogramming factors in order to avoid potential integration problems. The characterization of iTS-P cells derived from the RNA vector by methylation and microarray analyses clearly differed from that of ES cells or pancreatic islets. The advantages of iTS cells over iPS cells are (1) easier generation, (2) more efficient differentiation, and (3) no teratoma formation. Another group recently showed the induction of expandable tissue-specific stem/progenitor cells through the transient expression of YAP/TAZ^40^. The technology to generate iTS cells by reprogramming factors and tissue-specific selection may also be useful for the generation of other tissue-specific stem cells.

## Research Design and Methods

### Mice and cell culture

All mouse studies were approved by the review committee of University of the Ryukyus. The 24-week-old C57/BL6 mice (Charles River Laboratories Japan, Inc., Kanagawa, Japan) were used for primary pancreatic tissue preparations. Mouse pancreas was digested with 2 ml cold M199 medium containing 2 mg/ml collagenase (Roche Diagnostics Corporation, Indianapolis, IN, USA). The digested tissues were cultured in Dulbecco’s modified Eagle’s medium (DMEM; GIBCO-Invitrogen, Carlsbad, CA, USA) with 10–20% fetal bovine serum (FBS; GIBCO-Invitrogen). Eight-week-old nude or NOD/scid mice (CREA) were used for teratoma formation studies.

Mouse ES cells (ATCC, Manassas, VA, USA), iPS cells, and iTS cells were maintained in complete ES cell media with 15% FBS (Merck Millipore, Tokyo, Japan) on feeder layers of mitomycin C-treated STO or SNB cells, as described previously^1,41^. ES cells were passaged every three days, and iTS-P cells were passaged every five days.

### Generation of iPS and iTS-P cells by replicon transfection

iPS and iTS-P cells were generated as described previously^12^ using a Simpicom^TM^ RNA Reprogramming Kit (Millipore). Pancreatic cells were seeded onto a T25 plate on day 0 and cultured to 90–100% confluency on day 1. To minimize the IFN response, cells were treated with 1 mL Advanced DMEM containing 0.2 µg of B18R protein 2 h before transfection. A total of 1 µg RNA mixture (0.5 µg VEE-OKS-iG/0.5 µg B18R mRNA) was transfected with Lipofectamine 2000. After 3 h, the transfection medium was changed to Advanced DMEM containing 200 ng/mL of B18R protein. On day 11, Advanced DMEM was replaced with ES culture medium. Puromycin (0.8 µg/ml) was added from days 2 to 10. Cells were passaged onto STO feeder cells on day 11 and cultured in ES culture medium. Advanced DMEM containing 200 ng/mL of B18R protein was supplied every day until iPS or iTS-P colonies were generated.

### Quantitative PCR/RT-PCR

Total RNA was extracted from cells using an RNeasy Mini Kit (Qiagen, Tokyo, Japan). After quantifying the RNA by spectrophotometry, 2.5 µg of RNA was heated at 85 °C for 3 minutes and then reverse-transcribed into cDNA in a 25-µl solution containing 200 units of Superscript II RNase H-RT (Invitrogen), 50 ng random hexamers (Invitrogen), 160 µmol/l dNTP, and 10 nmol/l dithiothreitol. The reaction consisted of 10 minutes at 25 °C, 60 minutes at 42 °C, and 10 minutes at 95 °C. Polymerization reactions were performed in a Perkin-Elmer 9700 Thermocycler with 3 µl cDNA (20 ng DNA equivalents), 160 µmol/l cold dNTPs, 10 pmol appropriate oligonucleotide primers, 1.5 mmol/l MgCl_2_, and 5 units AmpliTaq Gold DNA polymerase (Perkin-Elmer, Norwalk, CT, USA) in 1X PCR buffer. The oligonucleotide primers are shown in Supplemental Table 1. The thermal cycle profile used a 10-minute denaturing step at 94 °C, followed by amplification cycles (1 minute denaturation at 94 °C, 1 minute annealing at 57–62 °C, and 1 minute extension at 72 °C) with a final extension step of 10 minutes at 72 °C.

The quantification of the mRNA was carried using the TaqMan real-time PCR system according to the manufacturer’s instructions (Applied Biosystems, Foster City, CA, USA). PCR was performed for 40 cycles, including 2 minutes at 50 °C and 10 minutes at 95 °C as initial steps. In each cycle, denaturation was performed for 15 seconds at 95 °C, and annealing/extension was performed for 1 minute at 60 °C. PCR was carried out in 20 µl of solution using cDNAs synthesized from 1.11 ng of total RNA. For each sample, the expression of mRNA was normalized by dividing by the Gapdh expression level. Primers for mouse insulin-1, insulin-2, Oct3/4, sex-determining region Y-box 2 (Sox2), Nanog, Foxa2, Sox17, hepatocyte nuclear factor 1β (Hnf1β), Hnf4α, Hnf6, Ptf1a, Ngn3, Sox9, Nkx6.1, the pancreatic and duodenal homeobox factor-1 (Pdx1), NeuroD, and Gapdh are commercially available (Assays-on-Demand Gene Expression Products; Applied Biosystems).

### Cell induction and differentiation

Directed differentiation into insulin-producing cells was conducted as described previously^21,22^, with minor modifications. ES cells (passage 45), iTS-P cells (passage 45) were used in this experiment. In stage 1, cells were treated with 25 ng/ml of Wnt3a and 100 ng/ml of activin A (R&D Systems, Minneapolis, MN, USA) in RPMI (Invitrogen) for 1 day, followed by treatment with 100 ng/ml of activin A in RPMI +0.2% FBS for 2 days. In stage 2, the cells were treated with 50 ng/ml of FGF10 (R&D Systems) and 0.25 µM of KAAD-cyclopamine (Toronto Research Chemicals, Toronto, Canada) in RPMI +2% FBS for 3 days. In stage 3, the cells were treated with 50 ng/ml of FGF10, 0.25 µM of KAAD-cyclopamine, and 2 µM of all-*trans* retinoic acid (Sigma-Aldrich, St. Louis, MO, USA) in DMEM +1% (vol/vol) B27 supplement (Invitrogen) for 3 days. In stage 4, the cells were treated with 1 µM of DAPT (Sigma) and 50 ng/ml of exendin-4 (Sigma) in DMEM +1% (vol/vol) B27 supplement for 3 days. In stage 5, the cells were then treated with 50 ng/ml of exendin-4, 50 ng/ml of IGF-1 (Sigma) and 50 ng/ml of HGF (R&D Systems) in CMRL (Invitrogen) +1% (vol/vol) B27 supplement for 3 to 6 days.

The differentiation of ES/iTS cells into insulin-producing cells was also conducted by EB/spheroid formation. To initiate EB/spheroid formation, a semi-confluent 10-cm plate of ES or iTS cells was harvested using trypsin, and cell clumps were resuspended in ES cell medium without LIF, allowed to aggregate, and transferred to one well of a nonadherent six-well plate. EBs/spheroids were allowed to undergo spontaneous differentiation for seven days in suspension, after which they were collected and taken for RNA and protein analyses.

### Teratoma formation/tumorigenicity assay

A total of 1 × 10^6^ to 1 × 10^7^ iPS/iTS cells were inoculated into each humerus and thigh of NOD/scid mice. As a positive control, we transplanted 1 × 10^6^ ES cells into 1 thigh of the NOD/scid mice.

### Immunostaining

The cells were fixed with 4% paraformaldehyde in PBS buffer. After blocking with 20% AquaBlock (EastCoast Bio, North Berwick, ME, USA) for 30 min at room temperature, the cells were incubated overnight at 4 °C with a guinea pig anti-insulin antibody (1:100; Abcam, Tokyo, Japan), rabbit anti-C-peptide antibody (1:200; Cell Signaling Technology, Danvers, MA, USA), goat anti-Pdx1 antiserum (1:100; R&D system, Minneapolis, MN, USA), or mouse anti-Nkx6.1 antiserum (1:100; Developmental Studies Hybridoma Bank, Iowa city, Iowa, USA) and then for 1 h at room temperature with FITC-conjugated anti-guinea pig IgG (1:250; Abcam), Alexa Fluor 647-conjugated anti-rabbit IgG (1:250; Cell Signaling Technology), NorthernLights^TM^ NL493-conjugated anti-goat IgG (1:200; R&D system, Minneapolis, MN, USA) or TRITC-conjugated anti-mouse IgG (1:200; Sigma-Aldrich). Mounting medium for fluorescence with DAPI (Vector Laboratories, Peterborough, UK) was used for mounting. The percentage of insulin/C-peptide-positive cells was calculated based on the ratio of immunostaining-positive cells/DAPI-positive cells in 12 visual fields.

### Insulin Release Assay

The insulin release was measured by incubating the cells in Functionality/Viability Medium CMRL1066 (Mediatech). The cells were washed three times in PBS and incubated in the solution (Functionality/Viability Medium CMRL1066) with 2.8 mM D-glucose six times for 20 min (total 2 hr) each to wash them. The cells were then incubated in the solution with 2.8 mM D-glucose for 2 hr, and then the solution with 20 mM D-glucose for 2 hr. The insulin levels in the culture supernatants were measured using an Ultra Sensitive Mouse Insulin ELISA (enzyme-linked immunosorbent assay) kit (Mercodia).

### Bisulfite genomic sequencing

Bisulfite treatment was performed using the CpGenome Turbo Bisulfite Modification Kit (Merck Millipore) according to the manufacturer’s recommendations. The PCR primers are listed in Supplemental Table 1. Amplified products were cloned using Mighty TA-cloning kit (TAKARA BIO INC, Shiga, Japan). Ten randomly selected clones were sequenced with the M13 forward and M13 reverse primers for each gene.

### Microarray

The total RNA from ES cells, iTS-P cells, or islets was labeled with biotin. Samples were hybridized to the GeneChip 3′IVT PLUS Reagent Kit (Affymetrix, Tokyo, Japan) and CeneChip Hybridization, Wash and Stain Kit (Affymetrix) according to the manufacturer’s protocol. Arrays were scanned with the GeneChip Scanner 3000 7G (Affymetrix). Data were analyzed using the Affymetrix GeneChip Command Console software program (Affymetrix).

### Statistical analyses

The data are expressed as the means ± standard error. Two groups were compared using Student’s *t*-test. P values of <0.05 were considered to indicate statistical significance.

All methods were performed in accordance with the relevant guidelines and regulations.

## Electronic supplementary material

Supplemental data
