# Supplementary material for: Characterization of induced tissue-specific stem cells from pancreas by a synthetic self-replicative RNA
Source: Sci Rep. 2018 Aug 17;8:12341. doi: 10.1038/s41598-018-30784-0 (PMC6098023; doi:10.1038/s41598-018-30784-0)
Supplement: Supplementary file 1 — Supplemental data [file 41598_2018_30784_MOESM1_ESM.docx]

**Original Article**

Characterization of induced tissue-specific stem cells from pancreas by a synthetic self-replicative RNA

Chika Miyagi-Shiohira ^1^, Yoshiki Nakashima ^1^, Naoya Kobayashi ^2^, Issei Saitoh ^3^, Masami Watanabe ^4^, Hirofumi Noguchi ^1,*^

1. Department of Regenerative Medicine, Graduate School of Medicine, University of the Ryukyus, Okinawa 903-0215, Japan

2. Okayama Saidaiji Hospital, Okayama 704-8192, Japan

3. Division of Pediatric Dentistry, Graduate School of Medical and Dental Science, Niigata University, Niigata 951-8514, Japan

4. Department of Urology, Okayama University Graduate School of Medicine, Dentistry and Pharmaceutical Sciences, Okayama 700-8558, Japan

**Supplemental Table 1. Primers**

Forward Reverse

R1 caggacgatctcattctcac gcttgccactcctctatcgtg

R2 ccacaatacgatcggcagtg atgtcctgcaacatattcaaa

R3 cggcgccagaagggcaagcg cacctgcttgacgcagtgtc

Gapdh accacagtccatgccatcac tccaccaccctgttgctgta

<methylation analysis>

Nanog p gattttgtaggtgggattaattgtgaattt accaaaaaaacccacactcatatcaatata

Oct3/4 p tggttgagtgggttgtaaggataggt actaacccatcacccccacctaata

Insulin-1 p tttagttaaagatgaagaaggt aaccacaaaaatactatttaac

Insulin-2 p ttttaggattaagtagaggtgttga aataaaataaaactcccaaaaaaaa

Pdx1 p gtaattttagaattggggaggaaaa accctaaaaccatcattaacctaaaa

Nkx6.1 p agttttagttaattaaaaggtgtgg ataaaaaaccaaaataaaaactttc

**SUPPLEMENTAL FIGURE LEGENDS**

**Supplemental Figure 1. Vector and time schedules for the generation of iTS-P cells from mouse pancreatic tissue.** (a) Schematic illustration of the VEE-RF RNA replicons. 5’ end nsP1–nsP4, nonstructural proteins 1–4; 3’ end reprogramming factors (Oct3/4, Klf4, Sox2, and Glis1). Location of 26S internal promoter, ribosome-shifting 2A peptide, IRES sequence, puromycin (Puro)-resistance gene, and PCR detection of replicon as indicated. (b) Time schedules for the induction of iTS-P cells. Mouse pancreatic tissue (1×10^5^ cells) was plated on day 0 and cotransfected (Tfx) with VEE-RF RNA replicon plus B18R mRNA on day 1. Selection by puromycin was performed from days 2 to 10. Cells were cultured in B18R-CM until day 18.

**Supplemental Figure 2.** Full-length gels of Figure 2c. No template was used as a negative control (NC). The VEE-RF RNA Replicon itself was used as a positive control (Vec). Size: R1=302bp, R2=302bp, R3=394bp, Gapdh=452bp
